# Advancing methane mitigation in ruminants through feed additive combinations: efficacy, interactions, and implementation challenges

**DOI:** 10.3389/fmicb.2026.1683471

**Published:** 2026-04-15

**Authors:** Eva Ramos-Morales, Juan Manuel Palma-Hidalgo, John Richard Newbold, Zaira Pardo, David R. Yañez-Ruiz, Charles James Newbold

**Affiliations:** 1 Estación Experimental del Zaidín, CSIC, Granada, Spain; 2 King’s Buildings, SRUC, Edinburgh, United Kingdom

**Keywords:** combinations, feed additives, hydrogen redirection, methane mitigation, ruminants

## Abstract

Feed additives have emerged as promising strategies to lower enteric methane (CH₄) emissions, with some achieving reductions of up to 30%. However, their widespread adoption is limited by inconsistent effects on productivity, regulatory barriers, and economic constraints. Combining feed additives with complementary modes of action might enhance mitigation outcomes, yet results vary depending on diet, adaptation period, additive interaction, and microbial dynamics in the rumen. This review synthesizes current knowledge on the efficacy, interaction effects, and implementation potential of feed additive combinations. It also examines economic incentives, such as carbon credit schemes, and recent policy developments that could accelerate adoption. While *in vitro* studies often show promising results, validation of combinations of feed additives in large-scale production systems is lacking. The complexity of hydrogen metabolism and microbial adaptation underscores the need for more integrated research efforts. Optimizing additive combinations that not only reduce CH₄ but also support animal productivity is essential for developing sustainable, climate-smart livestock systems.

## 1 Introduction

Climate change remains one of the most pressing global challenges, necessitating urgent action to reduce greenhouse gas (GHG) emissions. Among these, methane (CH₄) is particularly concerning due to its potent global warming potential—28 times that of CO₂ over 100 years. Given methane’s short atmospheric lifespan (approximately 12 years), reducing enteric CH₄ can yield relatively rapid climate benefits [Intergovernmental Panel on Climate Change (IPCC), 2021]. Enteric CH_4_ from ruminants accounts for 30% of anthropogenic CH_4_ and 5% of anthropogenic GHG emissions worldwide (Jackson et al., 2020). Given that 88% of livestock CH_4_ emissions arise from enteric fermentation, reducing these emissions is imperative to achieving the Paris Agreement’s goals of limiting global warming to well below 1.5 °C (Arndt et al., 2022). Moreover, at COP28, held in 2023, over 100 countries reaffirmed their commitment to reducing methane emissions by 30% by 2030 compared with 2020 levels (Global Methane Pledge, 2023). However, current mitigation efforts are insufficient to meet these goals, and substantial reductions in livestock methane emissions remain challenging due to the complex biological nature of rumen methanogenesis and the lack of widely adopted mitigation strategies.

Among available approaches, feed additives have emerged as one of the most effective mitigation strategies for reducing enteric CH₄ production (Almeida et al., 2021; Arndt et al., 2022). Several meta-analyses (Arndt et al., 2022; Beauchemin et al., 2022) have confirmed their potential to significantly lower CH₄ emissions, but their widespread adoption remains constrained by efficacy variability across diets and production systems, potential trade-offs with animal productivity, regulatory hurdles, and economic feasibility. This review briefly summarizes the main types of methane-inhibiting feed additives to provide essential context for understanding combinations. It also examines potential interactions when additives are combined and the role of scientific evidence in shaping their adoption within public and private incentive mechanisms.

## 2 Rumen methanogenesis and the role of feed additives

Enteric CH₄ production is a biological process driven by rumen methanogenesis, where hydrogen (H₂) from feed fermentation is primarily used to reduce CO₂ via the hydrogenotrophic pathway. This keeps ruminal H₂ levels low (Czerkawski, 1972; Ungerfeld, 2020) but results in CH₄ emissions, contributing to climate change and causing an energy loss of 3–10% of the animal’s gross energy intake (Niu et al., 2018). Inhibiting methanogenesis can lead to H₂ accumulation (Ungerfeld and Pitta, 2025), potentially disrupting fermentation if not efficiently redirected, as excess H₂ hinders NADH re-oxidation and impairs rumen metabolism (Wolin, 1981).

While redirecting reducing equivalents (2H) away from CH_4_ formation has the theoretical potential to enhance ruminant performance (Ungerfeld, 2018; Morgavi et al., 2023), current feed additives have not consistently delivered such benefits. When methanogenesis is inhibited, H₂ accumulates and increases the NADH/NAD^+^ ratio, limiting the capacity of rumen microbes to re-oxidise NADH. This constrains acetate formation and shifts fermentation towards alternative electron sinks such as propionate or butyrate (Ungerfeld and Pitta, 2025). Consequently, performance improvements may only emerge when CH_4_ inhibition is sufficiently strong to drive substantial redirection of H_2_ into energetically favourable pathways (Ungerfeld, 2022). Ni et al. (2025) demonstrated that 3-NOP-induced inhibition of methanogenesis triggers reductive acetogenesis as an alternative H_2_ sink, but the resulting metabolic gain is partially offset by H_2_ accumulation that simultaneously limits NAD + regeneration, which constrains the main acetate kinase route for VFA production and reduces the overall energy output of the microbial community. Similarly, a multi-omics study by Zhang et al. (2025) demonstrated that strong methanogenesis inhibition induces a broad restructuring of hydrogenotrophic pathways, with down-regulation of methanogenesis genes and up-regulation of alternative H₂-utilising pathways such as fumarate, nitrate, and sulphate reduction. An uncultured rumen bacterium (Dudenibacillus) was identified as a potential key driver of H_2_ redirection. Together, these findings underline the need for deeper mechanistic understanding to develop microbiome-based strategies that not only mitigate methane but also overcome metabolic constraints to enhance productivity.

In recent years, there has been a substantial number of general reviews, meta-analyses and reports evaluating methane-mitigating feed additives and management strategies (Almeida et al., 2021; Hegarty et al., 2021; Arndt et al., 2022; Beauchemin et al., 2022; Palangi and Lackner, 2022; Miller et al., 2023; Hodge et al., 2024; Hristov, 2024). According to the targeted microbes and metabolic pathways or processes affected, feed additives have different mechanisms of action on CH_4_ production (Belanche et al., 2025): (1) Modulation of the rumen microbial fermentation to decrease H_2_ production, (2) H_2_ redirection towards alternative electron (e-)-incorporating pathways, (3) Direct inhibition of methanogens and 4) CH_4_ oxidation, the latter being in a very initial stage of research (Table 1).

**Table 1:** Methane-mitigating feed additives: mode of action and barriers to adoption.

			Percentage reduction in methane yield (g/kg DMI)		Barriers to adoption				
Feed additive	Mode of action	Average dose	Beef	Dairy	Production improvements	Supply chain	Regulatory approval in EU	Delivery method	References
3-NOP^1^	Direct inhibition	70.5 mg/kg DMI	15%	31%	Milk fat and protein content (8%)	No residues	Authorised	Added to diet (TMR, premix etc.)	Yu et al. (2021), Kebreab et al. (2023)
Nitrate^2^	H2 acceptor		10%	10%	Not consistent	Toxicity over recommended dose	Registered feed material	In pelleted compound feed	Feng et al. (2020)
Blend of plant extracts^3^	Unclear	1 g/day		8.8%	Milk yield (3.6%), fat and protein content (4.1%), feed efficiency (4.4%)	No residues	Commercially available -Authorised as sensory feed additives	Added to diet (TMR, premix, etc.)	Belanche et al. (2020)
Asparagopsis genus	Direct inhibition	5 g/kg DMI	84.5%	29.5%	ADG in beef (10%)	Residues of bromine and iodine	Allowed as feed material	Added to diet (TMR)	Lean et al. (2021)
Monensin^4^	Increase in propionate	26.5 mg/kg DMI	2% (in g/d)	15.1% (in g/d)	None	No residues	Not authorised	Added to diet (TMR)	Appuhamy et al. (2013)
Garlic extract^5^	Direct inhibition	15 g/day	23%	29.5%	Milk yield (5%)	Garlic taste	Authorised as feed material / sensory feed additive	Pelleted	Roque et al. (2019), Vrancken et al. (2019)
Saponins	Protozoa inhibition	143 g/kg DMI	50%		Not consistent	Might be more impactful as grazing alternative	Commercially available	Added to diet (TMR) or grazing	Ku-Vera et al. (2020)
Condensed Tannins	Direct inhibition and increases propionate	7 g/kg DMI		17.7%	Suggests improved fermentability	Might be more impactful as grazing alternative	Commercially available	Added to diet (TMR) or grazing	Berça et al. (2023)

^1^Commercialised as Bovaer^®^, dsm-Firmenich GmbH, Kaiseraugst, Switzerland. ^2^Commercialised as SivAir^®^, Cargill Inc, Minneapolis, MN, USA. ^3^Commercialised as Agolin^®^, Agolin SA, Bière, Switzerland. Agolin SA was acquired by Alltech, Inc., Nicholasville, KY, USA, in May 2023. Agolin^®^ may be available under other brand names of local distributors in some countries. ^4^Commercialised as Rumensin^®^, Elanco Inc., Greenfield, IN, USA. ^5^Commercialised as EnterixTM, Mootral Holdings Ltd, Abertillery, UK.

The first group of CH_4_-reducing compounds includes microbial modulators, such as plant extracts and essential oil blends, which act by altering rumen fermentation to reduce H_2_ production. These compounds typically achieve only modest CH_4_ reductions (<10%; Hegarty et al., 2021). Other compounds that fall into this category, such as saponins, phenolics, condensed tannins, and fatty acids, have shown variable efficacy depending on the dose, source and underlying diet (Martins et al., 2024).

Among chemical H₂ sinks, nitrate is the only product that consistently reduces CH_4_ emissions (Feng et al., 2020). The commercial product SilvAir^®^ (calcium ammonium nitrate double salt) is classified as a feed material in the EU and can reduce methane emissions by over 20%; however, due to the risk of nitrite (NO₂^−^) accumulation and toxicity, inclusion levels are typically restricted to 1% of dry matter intake (DMI), achieving only a 10% reduction (Van Zijderveld et al., 2011). Microbial H₂ sinks offer promise by redirecting H₂ into metabolites like propionate or acetate, though optimal strains are still being identified (Li et al., 2024).

For targeting methanogens, 3-nitrooxypropanol (3-NOP, commercialized as Bovaer^®^), is the most effective additive authorized for use in dairy cattle in 60 countries worldwide, reducing CH_4_ by 25–30% at inclusion rates of 60–100 mg/kg DMI (Kebreab et al., 2023). Bromoform-containing macroalgae, such as Asparagopsis (taxiformis or armata), are even more effective, achieving CH_4_ reductions of 80–95% at inclusion rates of 0.5–2% of DMI. The use of Asparagopsis, however, is limited by concerns over bromoform’s potential carcinogenicity, environmental impact (ozone depletion) and stability, which have delayed its development and regulatory approval. A minimum bromoform concentration of approximately 12 mg bromoform/kg DMI is required for substantial CH_4_ reduction, and Angellotti et al. (2025) further showed that the efficacy of Asparagopsis declines as bromoform content decreases during storage, highlighting the critical importance of formulation stability.

**Figure 1:**
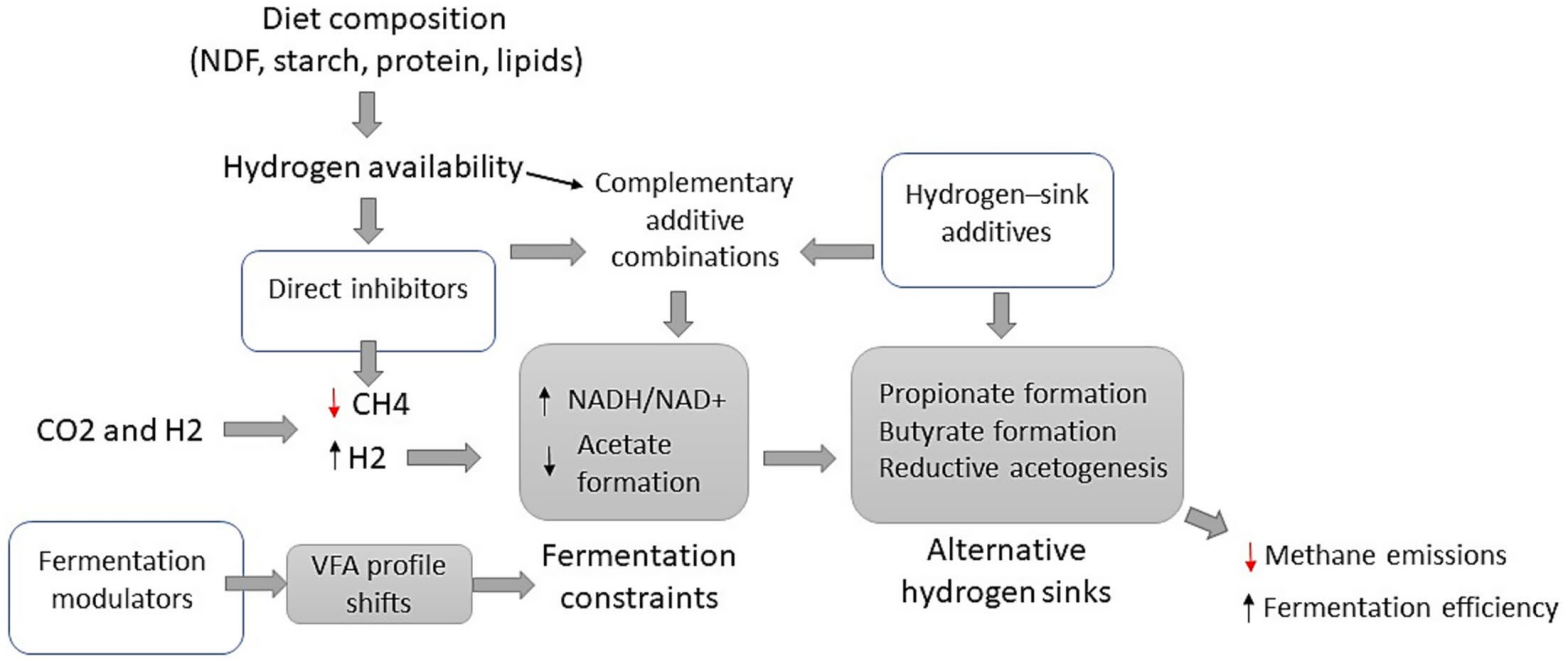
Conceptual framework illustrating how dietary context and complementary feed additive combinations influence hydrogen metabolism and methane mitigation in the rumen. Diet composition influences hydrogen availability during ruminal fermentation. Direct inhibitors suppress methane formation, resulting in reduced CH_4_ production and increased hydrogen accumulation. This shift can impose metabolic constraints on fermentation, reflected in increased NADH/NAD^+^ ratios and reduced acetate formation. Combining methanogenesis inhibitors with hydrogen-sink additives promotes hydrogen redirection toward alternative pathways such as propionate formation, butyrate formation or reductive acetogenesis. Fermentation modulators can further influence volatile fatty acid profiles and fermentation pathways.

The effectiveness of methane-mitigating additives is influenced by multiple factors, including dosage, diet composition, animal species, and production system. Diet composition in particular plays a key role because it determines baseline hydrogen availability in the rumen. Dietary fibre concentration (NDF) has been shown to influence the antimethanogenic response to 3-NOP (Dijkstra et al., 2018), while more recent reports indicate that dietary crude protein, starch and lipid concentrations can also moderate its efficacy (Maigaard et al., 2025; Dalmonte et al., 2026). These effects likely arise because diet composition affects ruminal hydrogen pressure and microbial activity, which determines whether inhibition of methanogenesis leads primarily to hydrogen accumulation or to its redirection toward alternative electron sinks. Similar diet-dependent variability has been reported for other additives, including plant secondary metabolites and nitrate sources. These metabolic constraints and context-dependent responses suggest that relying on single additives may not fully exploit the potential of methane mitigation strategies. Consequently, increasing attention has been directed towards combining additives with complementary modes of action, with the aim of simultaneously inhibiting methanogenesis and redirecting hydrogen towards alternative metabolic pathways. Figure 1 summarizes the conceptual framework linking diet composition, hydrogen availability, and complementary feed additive strategies that promote hydrogen redirection while mitigating methane emissions.

## 3 Combination of antimethanogenic feed additives

Although feed additives such as 3-NOP and Asparagopsis spp. can substantially reduce enteric CH_4_ emissions when used individually, relying on a single compound at high doses presents several limitations. The efficacy of these additives is dose-dependent but non-linear, meaning that increasing the dose does not proportionally enhance the mitigation effect (Mitsumori et al., 2012; Arndt et al., 2022). For example, high inclusion rates of asparagopsis may negatively affect feed intake due to bromoform content and low palatability (Muizelaar et al., 2021; Stefenoni et al., 2021). Reductions in DMI have also been reported when CH_4_ inhibition is strong enough to cause substantial H_2_ accumulation (Roque et al., 2019).

To evaluate combinations effectively, it is necessary to distinguish between independent and interdependent biological responses (Hristov et al., 2025). An additive effect is observed when the methane reduction obtained from a combination of additives equals the sum of the reductions achieved by each additive individually, suggesting that the additives act via distinct, non-competing pathways. Conversely, interactive effects (synergistic or antagonistic) occur when the observed response deviates from this mathematical sum, often due to overlapping roles in H₂ flux or shifts in the microbial community. However, interactions do not necessarily translate into greater overall mitigation than the additive prediction, as methane formation in the rumen is constrained by system-level factors including hydrogen flux, availability of alternative electron sinks, and microbial community adaptation.

Given the central role of hydrogen metabolism in rumen fermentation, feed additive interactions largely depend on how combinations influence hydrogen flows within the rumen ecosystem. Strategies that simultaneously inhibit methanogenesis and promote alternative hydrogen sinks are therefore theoretically complementary. However, the success of such combinations depends on the capacity of the rumen microbial ecosystem to redistribute excess reducing equivalents without disrupting fermentation. Consequently, the mitigation achieved by additive combinations is not determined solely by the individual mode of action of each compound but also by the metabolic constraints of the rumen environment. These constraints are strongly influenced by diet composition, as the type and availability of fermentable substrates regulate hydrogen production rates and fermentation pathways (van Lingen et al., 2016; Ungerfeld, 2020). In addition, host-related factors such as animal species can shape the rumen microbial community and its metabolic capacities, potentially leading to different fermentation patterns and hydrogen utilisation pathways even when the same substrate is provided (Li et al., 2022). These ecological and metabolic constraints partly explain why additive combinations do not always result in synergistic methane mitigation, even when their modes of action appear theoretically complementary. Table 2 summarises studies evaluating combinations of feed additives or feed ingredients with antimethanogenic potential and their effects on CH_4_ production or yield. Although oils are not classified as feed additives, they are included because of their frequent use in combinations studies and their demonstrated effects on CH_4_ production.

**Table 2:** Effect of different feed additives and ingredients, alone and in combination, on CH_4_ production (mL)/yield (g/kg DMI).

1st additive	%methane reduction 1st additive	2nd additive	%methane reduction 2nd additive	3rd additive	% methane reduction 3rd additive	Effect	Combined methane reduction^*^	Type of exp/Trial duration	References
Double combination									
Rumen modulator/oils + Alternative H2 sink									
Essential oils	~50%	Fumarate^2^	Not included in the design			Not determined	80%	Batch	Lin et al. (2012)
Saponin	12–25%	Nitrate	23–43%			Additive	32–58%	Batch	Patra and Yu (2013)
Monensin	20–39%	Nitrate (encapsulated)	22–24%			Additive	34–56%	RUSITEC	Capelari et al. (2018)
Saponin	~11%	Nitrate	33%			No effect	25%	Batch	Wu et al. (2019)
Tea saponins	0%	Nitrate	28%			No effect	28%	Dairy cattle −5 weeks	Guyader et al. (2015b)
Essential oils (microencapsulated)	0%	Nitrate (encapsulated)	13%			No effect	13%	Beef cattle −112 days	Alemu et al. (2019)
Sulfate	16%	Nitrate	32%			additive	47%	Sheep–4 weeks	Van Zijderveld et al. (2010)
MCFA (C10)	Not included in the design	Nitrate	17%			Not determined	40%	Batch	Vadroňová et al. (2023)
MCFA (10 y C12)	21%	Nitrate	9%			No effect	19%	Batch	Joch et al. (2023)
Canola oil	~9%	Nitrate	6%			Synergistic	25%	Sheep–21 days	Villar et al. (2020)
Linseed oil	17%	Nitrate	22%			Additive	32%	Dairy cattle–5 weeks	Guyader et al. (2015a)
Methane inhibitor + rumen modulator/oil									
3-NOP	70%	Monensin	12%			No effect	70%	RUSITEC	Romero-Pérez et al. (2016)
3-NOP	~40%	Monensin	0%			No effect	~40%	Beef cattle–105 days	Vyas et al. (2018)
3-NOP	~32%	Canola oil	~27%			Additive	51%	Beef cattle–10 weeks	Zhang et al. (2021)
3-NOP	28%	Canola oil	24%			Additive	51%	Beef cattle–28 days	Gruninger et al. (2022)
3-NOP	10%	Vitamin B12	10%			No effect	12%	Batch	Liu et al. (2023)
3-NOP	~10%	Cottonseed	~6%			Antagonist	No further reduction	Dairy cattle–8 weeks	Muñoz et al. (2024)
Methane inhibitor + alternative H2 sink									
3-NOP	~10%	Fumarate	~9%			No effect	11.50%	Batch	Liu et al. (2022)
Nitrate	20%	Fumarate	Not included in the design			No effect	~20%	Dairy cattle–7 days	Maigaard et al. (2024a)
Triple combination									
Saponin	~10%	Nitrate	~23%	Sulfate	~6%	Additive	46%	Batch	Patra and Yu (2014)
Saponin	29–36%	Nitrate	21–32%	Garlic	19–29%	Additive	68–71%	Batch	Patra and Yu (2015b)
Rapeseed oil	6–7%	Nitrate	12–13%	3-NOP	18–23%	No effect	23%	Dairy cattle−16 weeks	Maigaard et al. (2024b)

*Stating: represents initial batch culture; efficacy declined to 40.6% after 18 days of adaptation (Patra and Yu, 2015a).

Nitrate has been one of the most extensively evaluated additive in combination strategies, largely because of its role as an alternative hydrogen sink. As shown in Table 2, the response to nitrate-based combinations is highly variable, with outcomes being mostly additive (32–58% combined methane reduction, with a triple combination reaching 70% inhibition), or showing no detectable effect. Differences in the inclusion levels of inhibitors likely contribute to the inconsistent responses observed across studies. For example, *in vitro* studies by Wu et al. (2019) evaluated nitrate at 5 mmol L^−1^ whereas Patra and Yu (2013, 2014) tested levels up to 10 mmol L^−1^. Although the saponin dosage remained consistent across these studies (~0.6 g L^−1^), the lower nitrate inclusion in the former may have been insufficient to outcompete methanogenesis for available H_2_, failing to trigger the cumulative additive effects observed when the compounds were supplied at higher doses. Furthermore, variations in experimental substrate composition likely influence these outcomes; Patra and Yu (2013, 2014) utilized a balanced forage–concentrate diet (50/50 alfalfa hay to dairy concentrate), whereas Wu et al. (2019) employed a substrate high in dried distillers grains with solubles (DDGS). Differences in the starch-to-fibre ratio can alter microbial community structure and fermentation pathways, thereby influencing hydrogen production and the rumen’s capacity to buffer hydrogen accumulation (Romero-Pérez et al., 2016). Evidence from continuous culture systems further supports this diet dependency, as Capelari et al. (2018) reported that a nitrate–monensin combination reduced methane emissions by up to 56% in high-concentrate feedlot diets but only by 34% in dairy diets with higher forage inclusion. This suggests that the lower basal H_2_ pressure in high-concentrate diets may allow for more efficient redirection toward alternative sinks compared to fiber-rich diets where H_2_ production rates are higher.

Evidence from *in vivo* studies broadly supports the potential of nitrate-based combinations, although responses remain variable. Nitrate has been evaluated in combination with different dietary additives, including lipid supplements, saponins and essential oils (Table 2). Additive reductions in methane emissions have been reported when nitrate was combined with linseed oil (32%, Guyader et al., 2015a), while a synergistic effect was observed with canola oil (25% reduction, Villar et al., 2020) and no additional effect with medium-chain fatty acids (Joch et al., 2023). These results suggest that certain lipid profiles may complement nitrate’s H₂-sinking capacity by suppressing protozoal activity and reducing hydrogen availability for methanogenesis.

The consistent antimethanogenic efficacyt of 3-NOP has also prompted interest in combining this inhibitor with other additives or supplements that may redirect excess reducing equivalents or otherwise complement its mode of action. However, attempts to combine it with alternative hydrogen sinks or additives that modify fermentation pathways remain limited and inconsistent. Liu et al. (2022), in an *in vitro* study, showed that combining 3-NOP with fumarate reduced CH_4_ emissions slightly more than 3-NOP alone and alleviated H_2_ accumulation by redirecting it toward propionate production. While the authors interpreted this as a synergistic effect based on the regulatory impact on H_2_ flux, statistical interaction for CH4 reduction was not significant. Combinations of 3-NOP with rumen modifiers like monensin (Romero-Pérez et al., 2016; Vyas et al., 2018, or lipids (eg. canola oil Gruninger et al., 2022; Zhang et al., 2021) generally report no additional benefit. These findings suggest a metabolic saturation point: when 3-NOP is used at effective doses, it becomes the primary driver of CH_4_ reduction and the capacity of the rumen to further redistribute reducing equivalents is inherently constrained by the diet’s fermentation profile and the thermodynamic limits of the microbial community. Consequently, unless a secondary additive fundamentally alters the underlying metabolic constraints, the response to these combinations remains additive or is limited by the primary potency of 3-NOP.

## 4 Adaptation dynamics and long-term sustainability

The efficacy of additive combinations is intrinsically linked to the temporal adaptation dynamics of the rumen microbiota. Because the rumen is a highly resilient ecosystem characterized by functional redundancy, its response to combined additives is not static. For instance, fermentation modulators such as essential oils, and saponins, often require extended periods to induce stable shifts in microbial communities and fermentation patterns (Li et al., 2024), whereas nitrate requires a graduated transition to prevent nitrite accumulation and allow for the proliferation of nitrate-reducing bacteria (Villar et al., 2020). Conversely, direct inhibitors such as 3-NOP and Asparagopsis, act rapidly and do not require long adaptation periods.

These differing temporal dynamics imply that administering multiple additives simultaneously may hinder optimal microbial adaptation or overwhelm the rumen’s buffering capacity. The sequence of additive delivery could then be a critical factor in stabilizing these shifts. For example, establishing a robust alternative electron sink prior to methanogenesis inhibition may enhance hydrogen capture efficiency by ensuring the metabolic architecture for redirection is in place before hydrogen pressure peaks. Such sequential strategies aim to identify microbial signatures associated with a stable “low-methane, low-hydrogen” state.

However, even with optimized introduction strategies, a critical gap remains: the lack of long-term evaluation (>6 months) to ensure that initial mitigation gains are not undermined by microbial adaptation (Waters et al., 2025). While 3-NOP has shown persistent efficacy in longitudinal trials, the sustainability of combinations—where multiple metabolic pathways are simultaneously pressured—remains speculative. Consequently, it is essential to validate these combinations in living animals over their entire production cycle rather than relying on short-term data. Without long-term results, it is difficult to create reliable systems for reporting emissions in national inventories (del Prado et al., 2025). Future research must prioritize full-cycle trials to prove whether these strategies cause a permanent shift in the rumen’s microbial profile or if the ecosystem will eventually adapt and return to its original state.

## 5 Financial incentives and market opportunities for the use of antimethanogenic feed additives

Methane mitigating feed additives in farming introduce both costs and revenue opportunities. With lack of benefits for animal performance and relatively high costs, their long-term economic feasibility depends on carbon credit values, regulatory support, consumer readiness to pay premium prices, cooperative efforts across the industry to share costs equitably and direct payment schemes for farmers.

Carbon credit systems in the agricultural sector have increasingly attracted global attention as a tool to achieve climate neutrality. One carbon credit is equivalent to one metric ton of carbon dioxide equivalent (CO2e), a unit that combines the warming potential of CO_2_, CH_4_, and N_2_O. These systems allow agricultural producers to generate carbon credits by adopting practices that reduce GHG emissions. Credits can be sold to entities that need to offset their emissions, creating an opportunity for farmers to earn additional revenue. Carbon credit markets fall into two categories: compliance (mandatory) and voluntary. Compliance markets are regulated systems where industries or corporations are legally mandated to reduce their GHG emissions. If these entities exceed their emission limits, they are required to purchase carbon credits to compensate for their excess emissions. However, the *EU Emissions Trading System (EU ETS)* excludes most agricultural activities, and credits cannot currently be generated using methane-mitigating feed supplements, largely due to challenges in reliably measuring emissions reductions at the farm level (Verschuuren, 2022). Voluntary markets, meanwhile, serve businesses, organizations, and individuals voluntarily aiming to mitigate their carbon footprints, frequently driven by sustainability goals or corporate social responsibility commitments. Voluntary markets offer greater flexibility and have seen rapid growth, with platforms such as Gold Standard, Verra (VCS), and Soil Capital proactively supporting agricultural carbon credit initiatives.

Both Mootral and Agolin have demonstrated effectiveness in reducing CH_4_ emissions (Eger et al., 2018; Belanche et al., 2020), verified under Verra’s methodology VM0041, allowing the generation of tradable carbon credits known as Verified Carbon Units (VCUs) in voluntary carbon markets. While these carbon credits offer an additional income stream to farmers using antimethanogenic feed additives, their actual market value is often lower than that of credits under compliance schemes. As a result, the financial return may be insufficient to sustain long-term adoption. Farmers’ involvement in these schemes can be constrained by the high costs of accurate measurement, reporting and verification of emissions reductions. There is also a time lag between implementing mitigation practices and actual issuance and monetization of the carbon credits.

Including antimethanogenic feed additives in national GHG inventories is a crucial step towards ensuring that climate mitigation efforts in livestock systems are accurately measured and officially recognized. While this inclusion does not directly incentivize farmers, it provides the basis for policy tools—such as mandates or subsidies—that can support adoption. Denmark is the first country to fully commit to including antimethanogenic feed additives (authorized in the EU as zootechnical feed additives with a favourable effect on the environment) and feed ingredients like nitrate in its national GHG inventory. Other countries such as Netherlands, Spain and United States are at early stages focusing on data collection and methodology standardization.

## 6 Conclusions and future perspectives

Research on the development, validation and mode of action of feed additives to reduce rumen CH_4_ is, and will likely continue to be, a major focus in rumen microbiology in the coming years. While several effective additives are now reaching the market, their widespread adoption is hindered by the lack of a viable economic model—current carbon trading schemes are unlikely to fully offset implementation costs.

Achieving substantial and sustained reductions in enteric CH_4_ emissions will likely require moving beyond single inhibitors towards combinations of additives with complementary modes of action. In particular, strategies that simultaneously suppress methanogenesis while redirecting H₂ toward alternative pathways represent a promising direction. However, consistent and predictable hydrogen redirection has yet to be demonstrated under a wide range of dietary and production conditions. At the same time, advances in high-resolution functional microbiome approaches—including genome-resolved metagenomics, metabolic modelling, and emerging microbial engineering tools—may provide deeper mechanistic insight into hydrogen metabolism and microbial interactions in the rumen. Integrating these microbiome-level insights with nutritional strategies could help refine the design of additive combinations and support the development of durable and scalable methane mitigation solutions for ruminant production systems.
